# Transmissive silicon photonic dichroic filters with spectrally selective waveguides

**DOI:** 10.1038/s41467-018-05287-1

**Published:** 2018-08-01

**Authors:** Emir Salih Magden, Nanxi Li, Manan Raval, Christopher V. Poulton, Alfonso Ruocco, Neetesh Singh, Diedrik Vermeulen, Erich P. Ippen, Leslie A. Kolodziejski, Michael R. Watts

**Affiliations:** 1 Research Laboratory of Electronics, Massachusetts Institute of Technology, 77 Massachusetts Avenue, Cambridge, MA 02139 USA; 2 Department of Electrical and Electronics Engineering, Koç University, Rumelifeneri Yolu, Sariyer, 34450 Istanbul, Turkey; 3 John A. Paulson School of Engineering and Applied Science, Harvard University, 29 Oxford Street, Cambridge, MA 02138 USA; 4 Analog Photonics, One Marina Park Drive, Boston, MA 02210 USA; 5 Cambridge Graphene Centre, University of Cambridge, 9 JJ Thomson Avenue, Cambridge, CB3 0FA UK

## Abstract

Many optical systems require broadband filters with sharp roll-offs for efficiently splitting or combining light across wide spectra. While free space dichroic filters can provide broadband selectivity, on-chip integration of these high-performance filters is crucial for the scalability of photonic applications in multi-octave interferometry, spectroscopy, and wideband wavelength-division multiplexing. Here we present the theory, design, and experimental characterization of integrated, transmissive, 1 × 2 port dichroic filters using spectrally selective waveguides. Mode evolution through adiabatic transitions in the demonstrated filters allows for single cutoff and flat-top responses with low insertion losses and octave-wide simulated bandwidths. Filters with cutoffs around 1550 and 2100 nm are fabricated on a silicon-on-insulator platform with standard complementary metal-oxide-semiconductor processes. A filter roll-off of 2.82 dB nm^−1^ is achieved while maintaining ultra-broadband operation. This new class of nanophotonic dichroic filters can lead to new paradigms in on-chip communications, sensing, imaging, optical synthesis, and display applications.

## Abstract (web summary)

Optical filters are an integral part of many optical devices and circuits. Here, Magden et al. use a design based on mode evolution to demonstrate CMOS-compatible dichroic filters with more than an octave bandwidth, sharp roll-off and transmissive short- and long-wavelength outputs

## Introduction

Integrated optical filters are one of the most important and widely used building blocks in photonic systems. Many applications in optics utilize interferometric on-chip filters enabled by the silicon photonics technology including microring resonators^1–4^, Bragg or arrayed waveguide gratings (AWGs)^5–8^, contra-directional couplers^9,10^, and photonic crystal filters^11–13^ for in-band filtering. On the other hand, with recent advances in integrated supercontinuum^14–18^ and second/high-harmonic generation^19–23^, on-chip handling of ultra-broadband optical signals is becoming increasingly important. Being able to arbitrarily and effectively filter and combine hundreds-of-nanometers-wide signals is crucial for on-chip integration of key functionalities including *f* − 2*f*^24–26^ and *f* − 3*f*^17^ interferometry, spectroscopy^27,28^, and wideband wavelength-division multiplexing^29,30^. Yet, limited optical bandwidths of the aforementioned interferometric filters typically preclude their use in applications that require octave-wide bandwidths.

For general spectral combining or separation in ultra-broadband photonics, free-space dichroic filters are more suitable as they achieve bandwidths as wide as 1000 nm^31^. Interference within alternating layers of optical coatings with high index contrast allows dichroic filters to provide highly reflective, wideband, and flat-top pass-bands in short- or long-pass configurations. However, free-space dichroic filters are limited in scalability and are not suitable for optical systems requiring tens or hundreds of optical filters at precise wavelengths. Although integrated filters are better suited for such scalable and precise optical filtering, achieving similar flat-top responses in ring resonators^32^ or Mach–Zehnder interferometers^33^ results in higher insertion losses. Despite several examples of on-chip filters with stop-bands up to hundreds of nanometers^34,35^, an efficient spectral filtering device with transmissive, flat-top, and ultra-wideband short- and long-pass outputs is yet to be demonstrated.

Recently, various asymmetric couplers have been used for on-chip polarization and mode-division handling. Coupling between fundamental/higher-order modes^36,37^, transverse-electric (TE)/transverse-magnetic (TM) modes^38–40^, or combinations of higher-order and cross-polarization modes^41,42^ have been demonstrated. Waveguide asymmetry has also been achieved with the use of sub-wavelength gratings, where the refractive index is periodically modulated in the direction of propagation^43,44^ to create fiber-to-chip couplers^45^, waveguide crossings^46^, and polarization splitters^47^. Despite their typical use for polarization or mode-division purposes, similar asymmetric couplers can also be used in wideband spectral filtering by taking advantage of the wavelength dependence of evanescent field overlap^48,49^. In this coupling scheme, while mode-evolution of the propagating field allows for broadband operation, the coupling coefficient *κ* is primarily influenced by waveguide dispersion and therefore has a weak wavelength dependence. As a result, in contrast to the sharp spectral transitions in interferometric devices like ring resonators, AWGs, or Fabry–Perot cavities, these adiabatic spectral filters exhibit slow pass-band-to-stop-band transitions (estimated ~0.07 dB nm^−1^ in ref. ^48^ and ~0.04 dB nm^−1^ in ref. ^49^). Similar asymmetric Y-junctions have also been demonstrated in the past for wavelength separation, with the use of different waveguiding materials^50–52^ or sub-wavelength gratings^53,54^. Although most of these asymmetric coupler-based filters can separate wavelengths sufficiently far apart, they have not been able to simultaneously demonstrate an ultra-wideband response and a fast spectral roll-off. Therefore, an ideal integrated filter that combines the low-loss, broadband operation of adiabatic structures with the sharp spectral characteristics of interferometric devices is highly desirable.

In this article, we address the aforementioned challenges in optical filters by presenting the theory, design approach, and experimental results for broadband, low-loss, integrated, 1 × 2 port, transmissive dichroic filters that simultaneously achieve single-cutoff operation, octave-wide optical bandwidths, and sharp filter roll-offs. First, we introduce the concept of spectral selection in asymmetric waveguides by investigating possible cross-sections for spatial separation of guided modes. Then we model the transmission behavior in these waveguides using a coupled mode description. We show that a fast filter roll-off is expected when the individual propagation constants of two adjacent waveguides are matched at a single wavelength and are highly mismatched otherwise. This allows the cutoff wavelength to remain a separate design parameter independent of the propagation length along the coupler. We then design the transitions to the desired cross-sections, fabricate the devices in a standard complementary metal-oxide-semiconductor (CMOS) foundry, and experimentally verify the short- and long-pass filter characteristics. Demonstrated devices achieve low-loss operation and the sharpest filter roll-offs reported to date with octave-wide simulated bandwidth. Our results suggest that single-cutoff broadband filters created with the methods described here can be adapted in many integrated photonic platforms to create highly customizable and efficient optical devices such as band- or all-pass filters as well as ultra-broadband multiplexers and couplers.

## Results

### Coupled-mode description of spectral selectivity

In order to describe spectral selectivity in a given waveguide cross-section, we first analyze the wavelength dependence of field distribution in a system of two coupled waveguides: Waveguide A (WG_A_) and Waveguide B (WG_B_). Under the weak coupling assumption^55^, the guided modes of the combined structure (supermodes) can be approximated by linear combinations of the individual waveguide modes (*ψ*_A_ and *ψ*_B_ for WG_A_ and WG_B_, respectively). For waves carrying power in the same direction, the propagation of these supermodes can be described by a set of coupled differential equations^56^, which can be rewritten as

1 $$\left[ {\begin{array}{*{20}{c}} {\bar \beta + \delta } & { - \kappa ^ \ast } \\ { - \kappa } & {\bar \beta - \delta } \end{array}} \right]\psi _ \pm = \beta _ \pm \psi _ \pm$$

where *κ* is the coupling coefficient between the two waveguides, *β*_A_ and *β*_B_ are the individual propagation constants for *ψ*_A_ and *ψ*_B_ respectively, $\bar \beta = \left( {\beta _{\mathrm{A}} + \beta _{\mathrm{B}}} \right)/2$, and ${\delta=(\beta_{\mathrm{A}}-\beta_{\mathrm{B}})/2}$. The fundamental supermode, also known as the quasi-even mode, is described by the normalized eigenvector corresponding to the larger eigenvalue *β*_+_ and is expressed as

2 $$\psi _ + = \frac{1}{{\sqrt 2 }}\left[ {\begin{array}{*{20}{c}} { - \sqrt {1 + \delta /S} } \\ {\sqrt {1 - \delta /S} } \end{array}} \right]$$

where *S*^2^ = *δ*^2^ + |*κ*|^2 ^^57^. Once WG_A_ and WG_B_ are sufficiently separated, they can be treated as two outputs of an optical filter with their respective spectral transmissions. For the quasi-even mode input described above, the transmission for output port A, corresponding to WG_A_, is then the fraction of the optical power carried by *ψ*_+_ that remains in *ψ*_A_ given by

3 $$\left| {T_{\mathrm{A}}} \right| = \left| {\frac{{\psi _{\mathrm{A}} \cdot \psi _ + }}{{\psi _ + \cdot \psi _ + }}} \right|^2 = \frac{1}{2}\left( {1 + \frac{\gamma }{{\sqrt {1 + \gamma ^2} }}} \right) = \frac{1}{2}\left( {1 + {\mathrm{sin}}\left( {\mathrm{arctan}\,\gamma } \right)} \right)$$

where we defined the dimensionless quantity *γ* = *δ*/|*κ*|.

Equation (3) describes the distribution of power between any two coupled waveguides in general. Here the waveguide geometry can be engineered for the desired *δ* and *κ* or for a target spectral transmission. For spectral filtering and combining, we can exploit the wavelength dependence of *γ*. For instance, at the wavelength where *γ* = 0, power is evenly distributed between WG_A_ and WG_B_, marking the 3 dB cutoff where WG_A_ and WG_B_ are phase matched. In general, the sign of *γ* is determined by the magnitudes of *β*_A_ and *β*_B_. When *β*_A_ > *β*_B_, *δ* > 0, *γ* > 0, and |*T*_A_| > 1/2. As the difference *β*_A_ − *β*_B_ grows, *δ* becomes increasingly positive, |*T*_A_| approaches 1, and majority of the optical power in *ψ*_+_ is confined within WG_A_. As soon as *γ* drops below 0, the optical power in *ψ*_+_ is primarily confined in WG_B_, as *ψ*_+_’s spatial profile rapidly moves from WG_A_ to WG_B_. As a result, two coupled waveguides act as a spectral filter with a sharp transition and yet an extremely wide operation bandwidth.

**Fig. 1:**
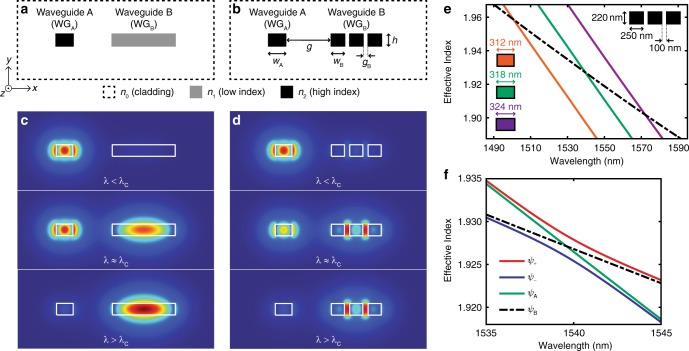
Spectrally selective waveguide cross-sections and their eigenmode analyses. **a** Waveguide cross-section created by two waveguides made from different core materials (*n*_0_ < *n*_1_ < *n*_2_). Waveguide A (WG_A_) is made from a higher index core than Waveguide B (WG_B_) and is therefore narrower to satisfy the phase matching condition at the cutoff wavelength (*λ*_C_). **b** Equivalent waveguide cross-section created using a single material where WG_B_ consists of closely spaced sub-wavelength core segments made from the same material as WG_A_. Fundamental transverse electric modes at wavelengths (*λ*) below, equal to, and above the cutoff *λ*_C_ **c** for the cross-section in **a** and **d** for the cross-section in **b**. With increasing wavelength, both sets of mode profiles indicate the spatial shift of the fundamental mode from WG_A_ to WG_B_. Owing to the design of the segmented WG_B_ in **b**, majority of the electric field is located between the segments, in the SiO_2_ cladding. Eigenmodes plotted with *n*_0_ = 1.45 for the SiO_2_ cladding, *n*_1_ ≈ 2.9 that can potentially be satisfied by a chalcogenide glass, and *n*_2_ = 3.48 for Si within the C-band. **e** Effective indices of WG_A_ (solid lines) and WG_B_ (dashed line) from the cross-section in **b**. Layer height *h* = 220 nm for both waveguides. Intersection marks the cutoff wavelength for each *w*_A_. The shift of cutoff toward longer wavelengths depends on the width change as well as derivatives of the plotted effective indices. **f** Effective indices for the quasi-even (*ψ*_+_) and quasi-odd (*ψ*_−_) supermodes and the individual guided modes in WG_A_ (*ψ*_A_) and WG_B_ (*ψ*_B_). Indices are calculated for *w*_A_ = 318 nm, and the same dimensions for WG_B_ as in **e**. The quasi-even supermode follows the highest effective index available in the cross-section

Consequently, the key to achieving a dichroic filter response is to use two waveguides that are only phase matched at the filter cutoff (*λ*_C_). The mismatch of propagation constants outside of *λ*_C_ is accomplished by waveguides with different group indices and is essential for a good extinction ratio between the two output ports. Two examples of these spectrally selective waveguide cross-sections are given in Fig. 1. Both examples consist of two waveguides whose guided modes are coupled via their evanescent fields. In Fig. 1b, WG_B_ possesses spectral characteristics equivalent to a waveguide with a smaller refractive index core, resulting in a smaller group index. This idea of mimicking a continuous waveguide using segments smaller than the wavelength is similar to a sub-wavelength grating. The difference here is that instead of using the grating duty cycle to create dissimilar waveguides^47,54^, we independently engineer the effective and group indices with greater flexibility by controlling the widths and separations of individual segments. Unlike many asymmetric couplers^36–42^, the phase matching condition here is satisfied between the fundamental TE modes of the two waveguides, instead of between TE/TM or fundamental/higher-order modes. As such, for the proposed designs, the group index difference is instrumental for realizing phase mismatch at wavelengths outside of the cutoff and therefore is key for a spectrally selective cross-section.

Major electric field components of TE quasi-even supermodes in Fig. 1c, d indicate the evolution of the mode profile between the two waveguides, as *γ* changes from positive to negative (see Methods). Since Eq. (3) describes power distribution in an arbitrary coupled waveguide geometry, any two waveguides satisfying *δ* = 0 at one wavelength can be used to create a similar spectrally selective waveguide cross-section. The design in Fig. 1b is more advantageous for silicon-on-insulator (SOI) platforms due to its single-material and single-step lithography process. For the rest of the analysis, we therefore focus only on this specific cross-section. In Fig. 1e, we plot the effective indices of the individual fundamental TE modes of WG_A_ and WG_B_ for this geometry. With a wider WG_A_, due to the larger propagation constant, the filter cutoff at which *β*_A_ = *β*_B_ shifts to longer wavelengths. The supermode effective indices are plotted in Fig. 1f, showing the evolution of the quasi-even supermode *ψ*_+_ from *ψ*_A_ to *ψ*_B_.

In principle, one can design WG_B_ with any number of core segments and with any widths and gaps: The ideal solution is a flat or bulk-like effective index profile that can maximize |*δ*| when *λ* ≠ *λ*_C_. One option is to use many core segments to mimic a bulk-like material, as the combination of closely spaced, sub-wavelength waveguides acts a single waveguide with a lower effective index. In reality, design choices are generally limited by the minimum widths and gaps that can be reliably fabricated in CMOS processes. Here we used a three-segment design to achieve a large *β*_A_ − *β*_B_ difference while ensuring reliable fabrication of individual segments.

### Power roll-off around the cutoff wavelength

**Fig. 2:**
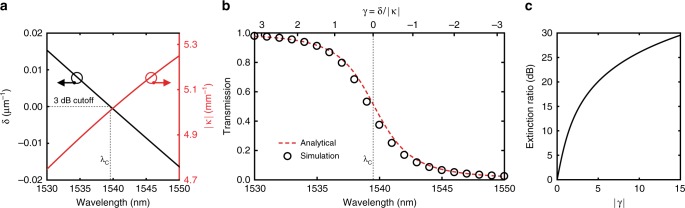
Power roll-off in the spectrally selective waveguide. **a** Effective index difference *δ* and the coupling coefficient *κ* as functions of wavelength (*λ*) around the desired cutoff in the C-band. At the 3 dB cutoff wavelength *λ*_C_, *δ* = 0 and *γ* = *δ*/|*κ*| = 0. For this specific design, *δ*(*λ* < *λ*_C_) > 0 and *δ*(*λ* > *λ*_C_) < 0. The wavelength-dependent change in the overlap and the coupling coefficient is primarily influenced by waveguide dispersion due to larger spatial profiles of individual waveguide modes *ψ*_A_ and *ψ*_B_ at longer wavelengths. Specifically, *κ* changes by about only 10% from 1530 to 1550 nm, whereas *δ* changes from positive to negative. **b** The expected transmission response |*T*_A_| at WG_A_, as a function of wavelength. With increasing wavelength, majority of the optical power shifts from WG_A_ to WG_B_, following the observed shift in the mode profile of *ψ*_+_. Analytical solution is calculated from the coupled mode analysis result in Eq. (3). Simulated results are obtained from ratios of Poynting vector fluxes through regions surrounding WG_A_ and WG_B_ to the total flux through the whole cross-section. Larger positive and negative values of *γ* correspond to transmissions closer to 1 or 0, on either side of the cutoff. **c** Ideal extinction ratio between the two output ports as a function of |*γ*| in the proposed waveguide cross-section. The extinction rapidly increases right after the cutoff and continues to increase slowly with growing |*γ*|

The evolution of power in the quasi-even mode from WG_A_ to WG_B_ depends on *γ*’s rate of change with respect to wavelength. As *γ* = *δ*/|*κ*|, *γ*’s wavelength dependence is determined by the change in the effective indices and the coupling coefficient as a function of wavelength. In Fig. 2a, we plot *δ*(*λ*) and |*κ*(*λ*)| for the waveguide cross-section in Fig. 1b, with a separation of *g* = 750 nm between WG_A_ and WG_B_. *δ*(*λ*) is highly dependent on wavelength and, by definition, undergoes a sign change around *λ*_C_. On the other hand, the coupling coefficient |*κ*(*λ*)| is only a slowly varying function of *λ*, as it mainly depends on the overlap of the evanescent fields of *ψ*_A_ and *ψ*_B_. The weak wavelength dependence of |*κ*(*λ*)| shown here explains the slow filter roll-offs for devices relying purely on waveguide dispersion. Since |*κ*(*λ*)| is monotonically increasing and only slowly varying, *γ*(*λ*) and *δ*(*λ*) exhibit similar spectral dependences. Therefore, the sign change in *γ*(*λ*) results in a maximum-to-minimum transition in |*T*_A_| where *γ*(*λ* = *λ*_C_) = 0 marks the 3 dB cutoff.

In Fig. 2b, we plot the expected transmission response |*T*_A_| for the cross-section in Fig. 1b. The analytical result plotted using the coupled mode solution in Eq. (3) shows excellent agreement with the simulated result obtained from ratios of Poynting vector fluxes. Using this transmission response, the expected extinction ratio between the two output ports is calculated as a function of |*γ*| and shown in Fig. 2c. As predicted, the extinction ratio grows rapidly with the initial increase of |*γ*|, as the optical mode shifts from one waveguide to the other. With subsequent increase of |*γ*|, the extinction ratio (in dB) only grows logarithmically, since most of the optical power is already contained within one waveguide.

The spectral change in |*T*_A_| towards 1 or 0 on either side of the cutoff depends on how fast the magnitude of *γ* can grow with respect to wavelength. As |*κ*(*λ*)| is only slowly varying, *γ*(*λ*)’s magnitude is mainly influenced by *δ*(*λ*). Therefore, in order to approach 1 or 0 transmission on the left or right of the cutoff, one must design WG_A_ and WG_B_ to achieve maximum effective index difference when *λ* ≠ *λ*_C_. Since the propagation constants *β*_A_ and *β*_B_ must match at *λ*_C_, the effective index difference for *λ* ≠ *λ*_C_ is only possible with waveguides with different group indices. With a larger group index difference, although a faster transition from 1 to 0 is expected, phase matching at the desired *λ*_C_ becomes increasingly challenging. One can also increase the magnitude of *γ* by separating WG_A_ and WG_B_ with a larger gap, effectively reducing *κ* for all wavelengths. Evolving the quasi-even mode to a final cross-section with a large WG_A_ − WG_B_ separation without losing power to other modes requires longer transitions. As a result, the separation of WG_A_ and WG_B_ presents a design trade-off between device length and performance.

### Adiabatic coupler design

**Fig. 3:**
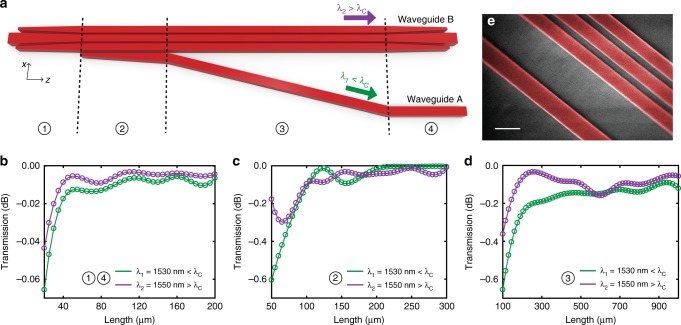
Optimization of adiabatic transitions and waveguide geometry. **a** Schematic illustration of short- and long-pass ports of the integrated dichroic filter with four transition sections to reach the desired waveguide cross-section. Spectrally selective cross-section is reached just before section ④, where wavelengths shorter than the cutoff (*λ*_1_ < *λ*_C_) and longer than the cutoff (*λ*_2_ > *λ*_C_) are separated to the short- and long-pass ports, respectively. (Si substrate and SiO_2_ buried and top oxides are not shown.) **b**–**d** Eigenmode expansion simulation results for quasi-even mode for determining the transition lengths for maximum transmission in sections ① and ④, ②, and ③. **e** A colorized scanning electron micrograph showing the fabricated waveguide cross-section. Scale bar, 400 nm

Once the appropriate waveguide geometry is determined for the desired spectral response, efficient transitions from a single-core rectangular waveguide to the determined cross-section must be designed. For low insertion-loss and a wide bandwidth, a single-mode TE input at any given wavelength must stay in the quasi-even mode throughout these transitions. According to coupled local-mode theory, power lost to unwanted modes can be minimized by decreasing the rate of change of the dielectric constant along the propagation length^58^, or equivalently, by using longer transitions^59^. To this end, we designed the slowly varying waveguide transitions illustrated in Fig. 3a for evolution of the quasi-even mode through the device. Transitions for conversion of a single waveguide input to a three-segment waveguide, development of a new adjacent waveguide segment (WG_A_), slow separation of the newly developed segment, and conversion of the three-segment structure back into a single rectangular waveguide have been implemented in sections numbered ① through ④, respectively. In all transitions, we used a 100 nm minimum waveguide width and spacing, as dictated by the fabrication design rules. For two sets of filters with cutoff wavelengths in the C-band and around 2100 nm, we simulated the transmission of the quasi-even mode through each section using the eigenmode expansion (EME) method (see Methods), as plotted in Fig. 3b–d. We chose *L*_1_ = *L*_4_ = 200 μm, *L*_2_ = 260 μm, and *L*_3_ = 900 μm in the C-band filters to minimize insertion loss while avoiding excessively long transitions to reduce losses due to propagation. Here, at the end of section ③, WG_A_ and WG_B_ have been separated to a final gap of *g* = 2 μm, in order to minimize any further coupling between the two waveguides and allow convenient connections to downstream devices. For the filters with cutoff wavelengths around 2100 nm, using the determined waveguide widths and gaps, appropriate coupling was obtained with lengths *L*_1_ = *L*_4_ = 200 μm, *L*_2_ = 350 μm, and *L*_3_ = 1100 μm through similar EME analyses.

In the proposed devices, performance metrics and the lengths of transitions ② and ③ heavily depend on the specific choices of the waveguide widths and gaps. While it may be possible to use a wider gap *g*_B_ for ease of fabrication, satisfying the phase matching condition at the same wavelength then requires either wider individual segments for WG_B_ or a narrower WG_A_. Wider segments in WG_B_ more strongly confine the propagating mode and reduce the coupling coefficient. As a result, an exponentially longer section ③ would be required to separate the propagating mode for *λ* < *λ*_C_, significantly increasing the device footprint. This may be mitigated by optimized adiabatic transitions with nonlinear tapers. On the other hand, compensating for larger *g*_B_ with a smaller *w*_A_ reduces the aspect ratio of WG_A_. This may be tolerable for filters with wider WG_A_ designed for use at longer wavelengths. However, for the C-band filter with *w*_A_ = 318 nm, reducing *w*_A_ further could potentially increase coupling to TM modes in the standard 220 nm SOI platform and result in a higher insertion loss at the short-wavelength output port.

**Fig. 4:**
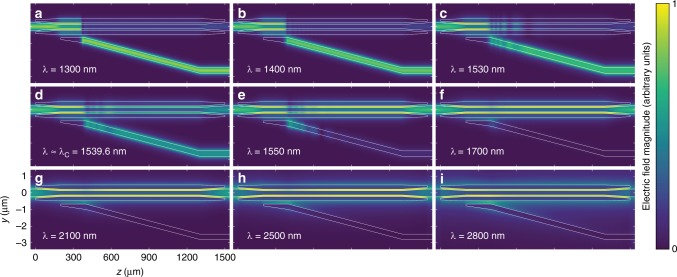
Simulated light propagation in the designed adiabatic transitions. **a**–**c** For wavelengths shorter than the cutoff (*λ* < *λ*_C_), majority of the input light reaches the bottom output port. **d** Input is evenly distributed between the two outputs at cutoff *λ*_C_ = 1539.6 nm. **e**–**i** Light remains in the top waveguide and is output through the top port for longer wavelengths (*λ* > *λ*_C_), demonstrating the estimated octave-wide bandwidth from 1300 to 2800 nm by eigenmode expansion simulations

Designed filters were fabricated in a standard CMOS fabrication facility (see Methods). A scanning electron micrograph of a fabricated device is shown in Fig. 3e where WG_A_ and the multi-segment WG_B_ are clearly resolved. For these fabricated filters with the dimensions above, we demonstrate the operation bandwidth in Fig. 4. Eigenmode expansion simulations at a wide range of wavelengths once again confirm that the input stays in the fundamental TE mode as it propagates through the designed couplers and is routed to the corresponding output port. Figure 4a, b show that the extinction ratio at short wavelengths is limited by *L*_2_, as majority of this short wavelength input couples to WG_A_ in section ②. This is in agreement with the inverse wavelength dependence of *κ*, confirming the need for a significantly larger *L*_2_ for the same ${\int}_{L_1}^{L_1 + L_2} \kappa \,\mathrm{d}z$ at shorter wavelengths. On the other hand, the extinction ratio at much longer wavelengths is limited by the final lateral separation of the two output waveguides. Figure 4g–i show the guided TE mode progressively widening with wavelength and increasing the amount of power coupled to WG_A_. With the above choices of lengths, widths, and gaps, the simulation results demonstrate the predicted octave-spanning operation bandwidth of the proposed filter from 1300 nm to over 2800 nm.

### Characterization and analysis of filter response

**Fig. 5:**
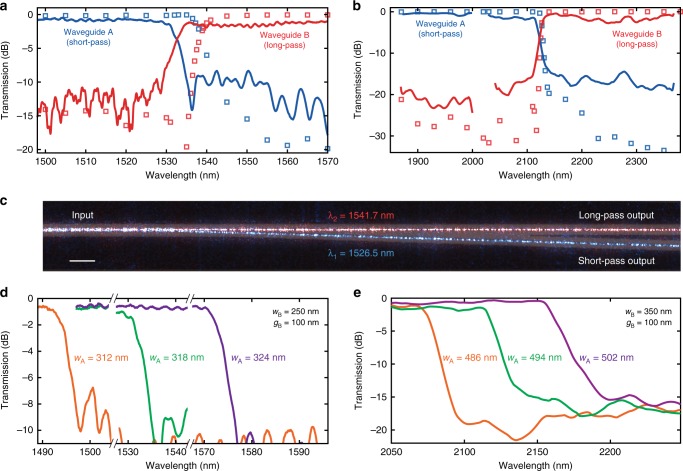
Measured and simulated spectral transmissions of dichroic filters. **a** Measurement (solid) and simulation results (squares) for the filter designed for C-band operation. The roll-off of power around the cutoff reaches 2.82 dB nm^−1^. Measured and simulated cutoff wavelengths are 1533.3 and 1539.6 nm, respectively. **b** A similar filter designed for operation around 2100 nm plotted with measured (solid) and simulated (squares) transmissions. Power roll-off for this longer wavelength filter is 0.64 dB nm^−1^ around the cutoff. Measured and simulated cutoff wavelengths are 2119.9 and 2128.6 nm, respectively. **c** Overlaid, colorized infrared images of light at wavelengths below and above the cutoff propagating through the dichroic filter and being separated to the respective output ports. Scale bar, 30 μm. **d** Shift of cutoff wavelengths for the C-band filter due to increasing *w*_A_ from 312 to 324 nm. **e** Similar shifts for the 2100 nm filter with *w*_A_ increasing from 486 to 502 nm. All eigenmode expansion simulated and measured cutoff wavelengths are summarized in Table 1

To test the fabricated filters, we used single-mode fibers, polarization controllers, and inverted facet tapers for on- and off-chip coupling of tunable, continuous-wave laser sources (see Methods). Figure 5a, b show the measured and simulated spectral responses of the filters. The measured spectrum at the short- and long-pass output ports confirm the predicted single cutoff response. The mismatch between the measurement and the design targets can be explained by the thickness and width variations due to fabrication. Since waveguide geometry influences propagation constants of the guided modes, minor differences between simulated and measured cutoff wavelengths are expected as in all integrated photonic filters. Operation of the C-band filter in Fig. 5a was captured by imaging the scattered light at wavelengths below and above the cutoff through a microscope objective. Overlaid images in Fig. 5c show the separation of the two inputs as they are routed to the short- and long-pass output ports.

The insertion loss through the filters in the pass-bands were measured to be approximately 1 dB for the short-pass ports and <2 dB for the long-pass ports. Higher loss in the long-pass output can be attributed to the increased interaction of the propagating mode with the sidewalls, due to the design of WG_B_. This sidewall interaction together with the coupling coefficient increase at longer wavelengths may also contribute to the slightly reduced extinction ratio in the long-pass output, due to scattering and coupling to the adjacent WG_A_. Extinction ratios of approximately 10 dB are measured for the C-band filter in Fig. 5a. For the 2100 nm filter in Fig. 5b, the measured extinction ratios were over 15 and 17 dB for the short- and long-pass ports, respectively. Power roll-off for the filters were calculated to be as high as 2.82 and 0.64 dB nm^−1^ by differentiating the transmission responses in Fig. 5a, b, respectively. Owing to the slowly varying transitions of waveguide cross-sections, the propagating mode in the dichroic filters demonstrated here does not rely on evanescent coupling to adjacent waveguides. Our measured roll-offs are therefore 10–70 times sharper than integrated filters relying on evanescent couplers^48,49^.

We can shift the cutoff wavelength by modifying the waveguide widths, which in turn changes the propagation constants in WG_A_ and WG_B_. As *δ*(*λ*) is modified, the cutoff wavelength at which *γ* = 0 is also shifted. This cutoff dependence on waveguide widths can be utilized to design and fabricate filters with different cutoff wavelengths. For instance, to shift the response to longer wavelengths, one can either increase *w*_A_, or increase *g*_B_, resulting in a higher *β*_A_ or a lower *β*_B_. Under both conditions, the wavelength at which *ψ*_A_ and *ψ*_B_ are phase matched shifts toward longer wavelengths. This spectral dependence on waveguide geometry is useful as it allows for adjustment of the cutoff without having to modify the adiabatic structures or the lengths *L*_1_ − *L*_4_.

To demonstrate the shift of the cutoff wavelength, a set of three filters were designed to have cutoffs around 1550 nm. In addition to nominal choice of *w*_A_ = 318 nm, filters with *w*_A_ = 312 nm and *w*_A_ = 324 nm were also fabricated. For this set of filters, we kept the previously chosen dimensions of WG_B_ constant at *w*_B_ = 250 nm and *g*_B_ = 100 nm. The expected cutoff shift is verified by measurements shown in Fig. 5d. Similarly, for longer wavelength applications, another set of three filters were designed in the 2100 nm spectral range, with *w*_A_ = 486 nm, *w*_A_ = 494 nm, and *w*_A_ = 502 nm. For this set of filters with longer cutoff wavelengths, we maintained the dimensions of *w*_B_ = 350 nm and *g*_B_ = 100 nm for WG_B_ and plotted the measured transmission data in Fig. 5e. For both cases, the EME simulations predicted similar extinction and roll-offs as before, at different cutoff wavelengths. It is important to note that, with these measurements, changes only in the width of WG_A_ are considered, while WG_B_’s width remains the same. For a correlated increase or decrease in the widths of WG_A_ and WG_B_, *β*_A_ and *β*_B_ would both change in the same direction. The resulting cutoff wavelength shift would then be much smaller and can be estimated from the derivatives of propagation constants with respect to widths and wavelength.

For both sets of filters, simulated and measured cutoff wavelengths are listed in Table 1 and show good agreement. The reason behind the slight mismatch is sensitivity of effective indices and the resulting phase matching point to fabrication variations, similar to many other silicon photonic devices. This is an inherent result of large derivatives of the propagation constants with respect to waveguide dimensions and is a common occurrence in high-index contrast platforms such as Si/SiO_2_ or SiN/SiO_2_. Here the difference between the simulated and measured cutoff wavelengths is about 5 nm on average and can be attributed to the photolithography-induced waveguide geometry changes. Many other devices that rely on phase matching also exhibit similar discrepancies as previously reported for Bragg gratings^5,6^, AWGs^7,8^, and contra-directional couplers^9^. For all these devices, precise spectral alignment is typically achieved with thermal tuning, as detailed in the following section. It may also be possible to accurately achieve the desired cutoff by designing WG_A_ and WG_B_ with group indices that are only slightly different, for instance, by using sub-wavelength gratings. The designed cross-section would then have a slowly changing |*γ*| as a function of wavelength and therefore a cutoff that does not depend on waveguide dimensions as much as before. The resulting trade-off between the filter roll-off and cutoff accuracy would need to be evaluated for the needs of the specific application.

**Table 1:** Summary of simulated and measured cutoff wavelengths for the fabricated dichroic filters

Waveguide dimensions			Filter cutoff (±0.1 nm)	
*w*_A_ (nm)	*w*_B_ (nm)	*g*_B_ (nm)	Simulated (nm)	Measured (nm)
312	250	100	1500.7	1495.1
318	250	100	1539.6	1533.3
324	250	100	1572.2	1573.9
486	350	100	2078.5	2076.3
494	350	100	2128.6	2119.9
502	350	100	2169.6	2162.4

*w*_A_, *w*_B_, and *g*_B_ refer to the waveguide widths and gaps depicted in Fig. 1b

### Thermal tunability of filter cutoff

The spectrally selective waveguide geometry allows for the cutoff wavelength to be thermally tuned, making use of the thermo-optic effect and thermal expansion in Si and SiO_2_. Here tunability is enabled by the imbalance in the thermal dependences of the effective indices of individual waveguide modes *ψ*_A_ and *ψ*_B_. According to eigenmode solutions at different temperatures, the temperature dependence of *β*_A_ is approximately twice as large as that of *β*_B_. This difference in thermal shifts arises from the respective geometries of the two waveguides: WG_A_ is a strip waveguide confining the majority of the field within its core. In contrast, WG_B_ is a slot waveguide^60^ where a significant amount of field remains in the SiO_2_ gaps between the waveguide cores. Together with SiO_2_’s much lower thermo-optic and thermal expansion coefficients than those of Si, this waveguide geometry explains the smaller thermal dependence of *β*_B_.

**Fig. 6:**
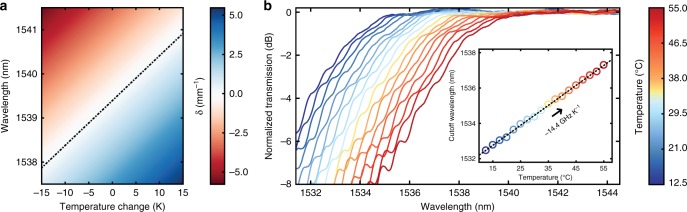
Thermal tunability of filter response. **a** Effective index difference *δ* plotted as a function of temperature change and wavelength, indicating the shift of cutoff wavelength with temperature. Dashed line traverses the cutoff wavelength for which *δ* = 0. **b** Measured, normalized transmissions demonstrating the cutoff shift with increasing temperature, as recorded at the long-pass output port of the dichroic filter. Inset shows linear fit to the cutoff wavelength as a function of temperature, yielding a thermal cutoff shift of −14.4 GHz K^−1^

In Fig. 6a, we analyze the cutoff wavelength dependence on temperature by plotting *δ* as a function of temperature and wavelength. For these simulations, we consider the thermo-optic and thermal-expansion effects for both waveguides (see Methods). The expected thermal dependence is calculated from the change of cutoff wavelength with respect to temperature and is equal to 101.5 pm K^−1^ or −12.8 GHz K^−1^. This is experimentally confirmed in Fig. 6b where we plot the normalized filter transmission at temperatures from 12.5 to 55.0 °C and the 3 dB cutoff at each temperature. From these cutoff wavelengths, the thermal shift was measured to be −14.4 GHz K^−1^. In the fabricated structures, the thermo-optic and the thermal expansion coefficients of the SiO_2_ cladding are dependent on the specific deposition parameters used. Together with the fabrication-induced waveguide geometry changes, any deviation in the thermal properties from the literature values used here^61,62^ may explain the mismatch between the simulated and measured thermal tuning efficiencies. As temperature is recorded at the substrate, thermal impedance of the buried oxide separating the waveguide and the substrate may also play a role in the difference.

The actual range of thermal tunability highly depends on the specific thermo-electric cooler (TEC) setup including the sizes of the TEC and the substrate, as well as the quality of the TEC–heatsink thermal contact. The measured tuning efficiency of −14.4 GHz K^−1^, however, confirms that demonstrated dichroic filters can be spectrally aligned to their design targets like many other systems with ring resonators^63–65^ or Bragg gratings^66,67^ with similar tuning efficiencies. The energy required for thermal tuning may be reduced with the use of microheaters directly integrated on top of sections ② and ③, using a process with metal heater layers. These integrated heaters would enable a larger Δ*T* and resulting Δ*λ* and also allow for multiple filters to be independently tuned.

## Discussion

The approach presented here provides a clear methodology to design highly selective, broadband, single-cutoff, transmissive, and low-loss optical filters based on the novel spectrally selective waveguide designs. These waveguides uniquely combine the broadband response of adiabatic transitions with the sharp spectral responses typically observed in interferometric filters. Since the transmission response in Eq. (3) depends on the propagation constants in the two waveguides, it is possible to design and integrate similar filters at visible or mid-infrared wavelengths using other core materials, such as Si_3_N_4_, Al_2_O_3_, or TiO_2_. Unlike devices that rely on propagation length to transfer power to an adjacent waveguide, the mode-evolution-based design here allows the cutoff wavelength to be determined independent of coupler length.

In theory, the proposed spectrally selective waveguide cross-section can provide much larger extinction ratios than measured with the fabricated devices, as predicted in Fig. 2c. From a design perspective, the measured extinction is determined by how effectively the input is evolved to the fundamental TE mode at the output. This explains the improved extinction ratio for the 2100 nm filters, due to their longer adiabatic transitions made necessary by their wider waveguides. The extinction ratio can be improved further by replacing the linearly tapered adiabatic sections ② and ③ with transitions optimized for a constant adiabaticity measure $\left\langle {\psi _{\mathrm{A}}\left| {\frac{\partial }{{\partial p}}} \right|\psi _{\mathrm{B}}} \right\rangle /\delta$ for a given parameter *p* such as the waveguide widths or gaps^57,68^. Specifically, section ② can be optimized for the width of WG_A_, which would include a nonlinear increase as it tapers up to its final width of *w*_A_. Similarly, the lateral separation *g* between WG_A_ and WG_B_ in section ③ can also be optimized, which would result in a faster increasing *g* as coupling reduces toward the end of the transition. These modified adiabatic couplers can greatly enhance coupling efficiency and extinction ratio while minimizing device length and suppressing fringing in the stop-bands. More generally, improvements may also be possible when using a cross-section similar to the one shown in Fig. 1a or by using lower index-contrast waveguides, due to reduced interaction of the guided mode with the sidewalls. Waveguide roughness may also contribute to the lower extinction ratio of the short-wavelength filter. Consequently, sidewall passivation, shorter transitions enabled by optimized adiabatic couplers described above, or the use of other waveguiding materials can also enhance the extinction ratio. However, material choices may be limited since phase matching at the cutoff wavelength poses strict restrictions on the material indices.

The mismatch between the measured cutoff wavelength and the design target is primarily due to fabrication-induced width and height changes that alter propagation constants, similar to other devices such as Bragg reflectors and ring resonators. More precise cutoff shifts may be possible with the use of wider waveguides at the expense of introducing higher-order modes, as the change in effective index gradually decreases with increasing width. Nevertheless, the expected and measured cutoff wavelength for varying *w*_A_ agree well. Additionally, thermal tuning of the cutoff wavelength is also demonstrated, as this is commonly required in reconfigurable optical systems. An accurately controlled wavelength shift is significant when designing band-pass filters that can be achieved by cascaded combinations of tunable short- and long-pass filters described here. Devices demonstrated in this article can be used in many optical systems including on-chip *f* − 2*f* and *f* − 3*f* interferometers, ultra-broadband multiplexing and demultiplexing systems in communications, efficient pump couplers to laser cavities, optical signal processing applications where arrays of arbitrarily wide band-pass filters are needed, and spectroscopy measurements that require spectral separation of excitation and output signals closely spaced in wavelength. Optical environmental sensors such as those used in index or concentration measurements or distance and velocity sensing like light imaging, detection, and ranging can also benefit from the filters described here, as they transduce wavelength selectivity to other measured quantities.

## Methods

### Finite difference eigenmode simulations

Waveguide modes and effective indices were simulated using the Lumerical’s MODE software with a maximum discretization of 10 nm. Finer mesh regions with 5 nm maximum discretization were used for the waveguides themselves. The coupling coefficient was calculated from the eigenvalues in Eq. (1) using 4|*κ*|^2^ = (*β*_+_ − *β*_−_)^2^ − 4*δ*^2^ and the propagation constants obtained from the eigenmode simulations. Transmission efficiencies plotted in Fig. 3b–d were extracted from the power transferred between the quasi-even input and quasi-even output modes as simulated by the EME method in MODE. In the direction of propagation, a total of 30 modes were solved every 5 μm in sections ① and ② and every 15 μm in region ③.

### Device fabrication

All filters were fabricated using 300 mm SOI substrates with a 225 nm-thick Si layer and a 2 μm-thick buried oxide in a CMOS foundry with 65 nm technology node. SOI was patterned using 193 nm immersion photolithography and reactive ion etching. The Si layer was fully etched to form the strip waveguides. This was followed by an oxidization step to passivate the sidewalls, which reduced the Si thickness to 220 nm. A 4 μm-thick top cladding of SiO_2_ was chemical vapor deposited. Dicing trenches were etched on the die borders, and the wafer was diced to individual dies.

### Optical characterization setup

Filters were characterized using three continuous-wave laser sources that are tunable from 1484–1625, 1860–2010, and 2040–2400 nm. Single-mode fibers SMF-28 or SM-2000 were used with a fiber polarization controller to couple TE light on- and off-chip through inverted facet tapers.

### Prediction and measurement of thermal shift

For temperature dependence, eigenmodes in WG_A_ and WG_B_ were solved for by modifying the refractive indices and dimensions of the core and cladding materials using the following thermo-optic and thermal expansion coefficients: d*n*_Si_/d*T* = 1.86 × 10^−4^ K^−1^^ 69^, d*n*_SiO2_/d*T* = 0.95 × 10^−5^ K^−1^^ 61^, *α*_Si_ = 2.61 × 10^−6^ K^−1^^ 70^, and *α*_SiO2_ ≈ 5 × 10^−7^ K^−1^^ 62^. In addition to the 5 nm meshes, edges of the waveguides and the gaps in between were spatially discretized with 2 nm resolution to accurately capture the small changes in the refractive index as a function of temperature. The simulation was centered at the free-running operation temperature of *T*_0_ = 22.6 °C as measured by the TEC with ~1 mW optical input power. Eigenmode simulations were performed with wavelength and temperature steps of Δ*λ* = 0.5 nm and Δ*T* = 1 K, respectively. For measurement, the temperature was controlled by using a closed-loop feedback-driven TEC placed in contact with the chip substrate.

### Data availability

The data that support the findings of this study are available from the corresponding author upon reasonable request.

## Electronic supplementary material

Peer Review Report
