# Supplementary material for: Transmissive silicon photonic dichroic filters with spectrally selective waveguides
Source: Nat Commun. 2018 Aug 1;9:3009. doi: 10.1038/s41467-018-05287-1 (PMC6070617; doi:10.1038/s41467-018-05287-1)
Supplement: Supplementary file 1 — Peer Review Report [file 41467_2018_5287_MOESM1_ESM.pdf]

Reviewers' comments:

Reviewer #1 (Remarks to the Author):

This paper demonstrated a highly selective, transmissive optical filters with sharp roll-offs, which is interesting for some applications. The theory, design, and experimental characterization of the optical filters are presented. The structure is novel and the performance is impressive. There are some comments given below.

1. In recent years asymmetric directional couplers on silicon have been very popular for on-chip polarization-handling as well as mode-coupling by manipulating the phase-matching condition between the two or more waveguide in the coupling region. It might be necessary for the authors to give a summary for the related work reported previously.
2. It is indeed very important to achieve high-performance on-chip pass-band optical filters, e.g., for WDM systems. On the other hand, dichroic filters are very popular for free-space optical setup. It might be necessary to talk more about the motivation of developing on-chip dichroic filter. What are the potential applications?
3. About the fabrication tolerance. As shown in Fig. 4, the transition wavelength is very sensitive to the waveguide dimension. On the other hand, the present design is based on an adiabatic taper, which is basically insensitive to the dimensional variation. Is it due to the shift of the phase-matching point? Please given an explanation. Thermal-tuning might help a little. However, the tenability range is still very limited. How to achieve the wavelength alignment accurately?
4. The extinction ratio for the transmissions shown in Fig. 4(d) is about 10dB, which is pretty low for optical filtering. In contrast, the extinction ratio for Fig. 4(d) is higher than 15dB, which is more decent. Please give a discussion about how to achieve higher extinction ratio.
5. This device is designed with a 100nm minimum waveguide width and spacing. This nano-scale size can be fabricated with E-beam litho process. However, it is usually not allowed for deep-UV litho. Please give a discussion about the feature size. Is it possible to choose larger gap/tip widths for the design, e.g., 150nm or 180nm? How about the performance?
6. It will be helpful for readers if the authors show the simulated light propagation in the designed device.

Reviewer #2 (Remarks to the Author):

The paper entitled “Transmissive, Silicon Photonic Dichroic Filters with Spectrally Selective Waveguides,” By E. Magden et. Al presents the use of waveguides for wavelength selectivity. These serve as single wavelength cutoff filters. The work is interesting and sound and thorough, providing theory, experimental verification. However, the performance metric they use of 2.82dB/nm roll-off is not particularly impressive even within some of the devices cited in Refs. 19-29. Other types of filters which also utilize coupled mode theory in their design and description have been demonstrated rather widely with better roll-off figures. Though not described as dichroic filters, these other filters also serve a similar purpose, which is wavelength selection. Examples of such filters can be broadly classified as mirrors, AWGs, add/drop filters, photonic crystals, wavelength division multiplexers, mode division multiplexers, Bragg gratings (Refs. 19-29 in the manuscript and the following):

Y. Wang et. al, “Ultra-broadband compact and high-reflectivity circular Bragg grating mirror based on 220nm silicon-on-insulator platform,” Opt. Express 25(2017). – Few hundred nanometer stopband
J. Foresi et. Al, “Photonic-bandgap microcavities in optical waveguides,” Nature 390 (1997) – 300nm stopband

One possible argument in favor of the present device could be that the design presented is a pure

cutoff filter which rejects anything outside of the cutoff wavelength, rather than possessing just a limited range of rejected or transmitted wavelengths. However, there are filters with transmission or rejection bandwidths of hundreds of nanometers, which make them functionally similar to the Dichroic filters in this work for most applications. Photonic crystals, AWGs, or grating based filters can also be designed to be non-FSR limited with sufficiently large bandwidth to essentially behave as quasi-single wavelength cutoff filters.

At the 2100nm wavelength, the transmission bandwidth demonstrated by the authors spans about 200nm. Ref. 2 above is a photonic-bandgap microcavity with a bandwidth of 300nm which is somewhat larger. One suggestion is for the authors to include some simulation results on the potential wavelength span that the long pass and short pass regions can possess, as it is not always straightforward to characterize due to the need for multiple broadband sources.

The narrative within the introduction needs improvement. For example:

1) "Many applications in wavelength division multiplexing (WDM) require sharper cutoffs for more efficient utilization of the available spectrum." – WDM in silicon photonics widely demonstrate better cutoffs already, and WDM applications would benefit from actual wavelength selectivity within a narrow range and not a single cutoff.

2) The same can be said of this sentence, "However, narrow pass-bands or limited operation bandwidths present challenges in ultra-wideband photonics as well as WDM applications where many channels have to be squeezed into a narrow spectrum," where WDM applications typically adhere to the 100GHz or 200GHz standard and would need spectral selectivity over a limited wavelength range rather than a single wavelength cutoff. What is left as a potential application is mentioned as ultra-wideband photonics, but the authors do not give good examples of this. Consequently, the authors could have written a much stronger introduction and found a much better problem statement.

In its present form, I do not recommend publication in Nature Communications as the advancement over the performance of existing devices is not significant, and this has not been well articulated. In particular, the way it is written to emphasize the roll-off value (2.82dB/nm) which is not spectacular in the domain of silicon-based wavelength filters. Having said that, if the authors can demonstrate some other new metrics or functions associated with their devices that are significantly better than existing filters, it could warrant reconsideration for publication Nature Communications.

Reviewer #3 (Remarks to the Author):

• The paper presents a novel approach to creating integrated photonic spectral filters, with highly desirable high-pass & low-pass cut-off filters, with very sharp roll-offs both in predictions and experimental results. If the problems can be addressed, I expect that it may have a large impact in the field, where many applications of this idea can be applied to.
• Specifically, the major limitation is that the extinction ratio is only 10 dB for the 1550 nm devices, where typically > 30 dB is required for practical applications, including the ones mentioned in the discussion section. At minimum, the manuscript should provide a design (not necessarily fabricated) that solves this issue.
• There is some similarity of this work to previous publications, specifically in regards to Fig 1a/b, where WGb is a sub-wavelength waveguide (along the propagation direction, versus in the transverse direction), and where the concept of phase matching between a solid waveguide and a sub-wavelength waveguide is used. The authors should refer their discussion on phase matching of dissimilar waveguides to previous publications. The following was a directional coupler-based

designs, rather than adiabatic, hence is not the same device as this submission: https://www.osapublishing.org/ol/fulltext.cfm?uri=ol-39-24-6931&i d=306213. And there are other works on polarization conversion using strip to SWG converters.

• The device is highly sensitive to fabrication variations (width, thickness of waveguides). How does it compare to other approaches (AWG, Bragg gratings, contra-directional couplers, etc)?

• On Page 5: "How fast the power in the quasi-even mode evolves from WGA to WGB depends on γ's rate of change with respect to wavelength." Does "how fast" refer to the time domain, or equivalently the propagation distance? Or with respect to wavelength? This needs clarification / correction. Similar comment for "The speed at which …" a few paragraph later.

• The discrepancy between the simulated versus measured thermo-optic tuning coefficients is almost 20%, which is a much larger error than the cut-off wavelength discrepancy (5 nm at 1550) which is only 0.3%. Yet, the same explanation is given for both discrepancies. Clearly there much be more to the story than just a slight difference in the fabricated thicknesses and widths. More investigation and explanation is required.

• The transmission spectra of the measured devices should be overlaid with the simulated devices.

• The manuscript is sufficiently detailed for others to be able to reproduce the work.

We thank the reviewers and the editors for their constructive comments and suggestions. We have revised the manuscript accordingly and have provided detailed responses to each comment below.

Legend
Reviewers' comments
Author responses and revisions made in the text including additions
Direct quotes from the revised manuscript

Reviewer #1 (Remarks to the Author):

This paper demonstrated a highly selective, transmissive optical filters with sharp roll-offs, which is interesting for some applications. The theory, design, and experimental characterization of the optical filters are presented. The structure is novel and the performance is impressive. There are some comments given below.

1. In recent years asymmetric directional couplers on silicon have been very popular for on-chip polarization-handling as well as mode-coupling by manipulating the phase-matching condition between the two or more waveguide in the coupling region. It might be necessary for the authors to give a summary for the related work reported previously.

The reviewer correctly indicates that the idea of using dissimilar waveguides is similar to some of the work on asymmetric directional couplers used for TE/TM or fundamental/higher-order mode coupling. As also suggested by reviewer #3, sub-wavelength gratings are also used in similar asymmetric couplers. We summarized this work in paragraph 3 of the introduction by including the following details on numerous types of asymmetric couplers:

"Recently, various asymmetric couplers have been used in on-chip polarization or mode-division handling. Coupling between fundamental/higher-order modes [36,37], transverse-electric/transverse-magnetic modes [38-40], or combinations of higher-order and cross-polarization modes [41,42] have been demonstrated. Waveguide asymmetry has also been achieved with the use of sub-wavelength gratings, where the refractive index is periodically modulated in the direction of propagation [43,44] to create fiber-to-chip couplers [45], waveguide crossings [46], and polarization splitters [47] … Similar asymmetric Y-junctions have also been demonstrated in the past for wavelength separation, with the use of different waveguiding materials [50-52] or sub-wavelength gratings [53,54]. …"

We also referred our phase matching discussion in our first Results section to this summary we provided in the introduction. We identified the key similarities and differences, and added the following explanation in paragraph 2 of page 4:

"…This idea of mimicking a continuous waveguide using segments smaller than the wavelength is similar to a sub-wavelength grating. The difference here is that instead of using the grating duty cycle to create dissimilar waveguides [47,54], we independently engineer the effective and group indices with greater flexibility by controlling the widths and separations of individual

segments. Unlike many asymmetric couplers [36-42], the phase matching condition here is satisfied between the fundamental TE modes of the two waveguides, instead of between TE/TM or fundamental/higher-order modes. As such, for the proposed designs, the group index difference is instrumental for realizing phase mismatch at wavelengths outside of the cutoff, and therefore is key for a spectrally selective cross-section. ”

2. It is indeed very important to achieve high-performance on-chip pass-band optical filters, e.g., for WDM systems. On the other hand, dichroic filters are very popular for free-space optical setup. It might be necessary to talk more about the motivation of developing on-chip dichroic filter. What are the potential applications?

In recent years, the integrated photonics community has witnessed significant advances in ultra-broadband photonics including supercontinuum generation [14-18] at various parts of the spectrum as well as second/high harmonic generation [19-23]. Being able to arbitrarily and effectively filter and combine hundreds of nanometers wide signals is crucial for on-chip integration of key functionalities including f-2f [24-26] and f-3f [17] interferometry, spectroscopy [27,28], or wideband WDM [29,30]. Yet, limited optical bandwidths of the aforementioned interferometric filters typically preclude their use in applications that require octave-wide bandwidths.

Therefore, an on-chip dichroic filter like the one presented in the article can enable full integration of the above applications, by bringing broadband efficient wavelength selectivity to integrated systems. We have added these details in the first paragraph of the introduction to better motivate the device and more clearly explain this missing on-chip functionality.

3. About the fabrication tolerance. As shown in Fig. 4, the transition wavelength is very sensitive to the waveguide dimension. On the other hand, the present design is based on an adiabatic taper, which is basically insensitive to the dimensional variation. Is it due to the shift of the phase-matching point? Please given an explanation.

The filter cutoff is determined by the waveguide cross-section itself (Fig 1b), and is indeed the wavelength at which WG_A and WG_B are phase matched. The reason for using adiabatic couplers is to have a large optical bandwidth, i.e. to make sure that optical input at a wide range of wavelengths can be evolved through the couplers to the corresponding mode shape in Fig 1d. The devices with wider WG_A in Fig 5 (previously Fig 4) are designed to demonstrate precisely what the reviewer said: the shift in the phase matching point (filter cutoff) to longer wavelengths, but only as a function of WG_A's width, and with the purpose of intentionally shifting the cutoff wavelength. Dimensions of WG_B were kept constant in these devices; and therefore, β_B remains the same. In contrast, for a correlated increase in the widths of both WG_A and WG_B, since effective indices of both ψ_A and ψ_B increase, there would be a smaller shift in the cutoff wavelength, depending on the derivatives of β_A and β_B with respect to width and wavelength. This is also why any correlated width changes (possibly due to fabrication) in the two

waveguides affect the cutoff much less than an uneven change in one of the waveguides. Therefore, despite the fabrication dependence of the device, there is good agreement between the simulated and measured cutoff wavelengths, which are off by about 5 nm on average. We clarified these details by adding the above explanation in paragraphs 3 and 4 of the "Characterization and analysis of filter response" section.

In paragraph 5 of the same section, (since reviewer #3 also asked) we provide a comparison of the fabrication tolerance with respect to other resonant filters: "The reason behind the slight mismatch is sensitivity of effective indices and the resulting phase-matching point to fabrication variations, similar to many other silicon photonic devices. This is an inherent result of large derivatives of the propagation constants with respect to waveguide dimensions, and is a common occurrence in high index contrast platforms such as Si/SiO_2 or SiN/SiO_2. Here, the difference between the simulated and measured cutoff wavelengths is about 5 nm on average, and can be attributed to the photolithography process-induced changes waveguide geometry. Many other devices that rely on phase-matching also exhibit similar discrepancies as previously reported for Bragg gratings [5,6], AWGs [7,8], and contra-directional couplers [9]. For all these devices, precise spectral alignment is typically achieved with thermal tuning, as detailed in the following section…"

Thermal-tuning might help a little. However, the tunability range is still very limited. How to achieve the wavelength alignment accurately?

"The actual range of thermal tunability highly depends on the specific TEC setup including the sizes of the TEC and the substrate, as well as the quality of the TEC-heatsink thermal contact. The measured tuning efficiency of −14.4 GHz/C however confirms that demonstrated dichroic filters can be spectrally aligned to their design targets like many other systems with ring resonators [63–65] or Bragg gratings [66, 67] with similar tuning efficiencies. The energy required for thermal tuning may be reduced with the use of microheaters directly integrated on top of sections ② and ③, using a process with metal heater layers. These integrated heaters would enable a larger ΔT and resulting $\Delta\lambda$, and also allow for multiple filters to be independently tuned." We expanded the "Thermal tunability of filter cutoff" section by adding the details above in paragraph 3.

A design-based solution for achieving accurate cutoff wavelength would be to design WG_B with a group index that is not too far off from that of WG_A. This is easily achieved with the use of a sub-wavelength grating. This results in a $|\gamma|$ that does not change very rapidly with changing wavelength, and consequently, a cutoff wavelength that does not depend as highly on the waveguide widths as before. However, this will also result in a slower filter roll-off. This may be a tolerable tradeoff, depending on the specific application. We also added these details at the end of paragraph 5 in the "Characterization and analysis of filter response" section.

4. The extinction ratio for the transmissions shown in Fig. 4(a) is about 10dB, which is pretty low for optical filtering. In contrast, the extinction ratio for Fig. 4(d) is higher than 15dB, which is more decent. Please give a discussion about how to achieve higher extinction ratio.

We added a new paragraph (Discussion, paragraph 2) about the extinction ratio and outlined various design improvements that will significantly improve the extinction for future devices. This paragraph is copied below:

"In theory, the spectrally-selective waveguide cross-section can provide much larger extinction ratios than measured with the fabricated devices, as predicted in Fig 2c. From a design perspective, the measured extinction is determined by how effectively the input is evolved to the fundamental TE mode at the output. This explains the improved extinction ratio for the 2100 nm filters, due to their longer adiabatic transitions made necessary by their wider waveguides. The extinction ratio can be improved by replacing the linearly tapered adiabatic sections ② and ③ with transitions optimized for a constant adiabaticity measure $\left\langle \psi_A \middle| \frac{\partial}{\partial p} \middle| \psi_B \right\rangle / \delta$ for a given parameter p such as the waveguide widths or gaps [57, 68]. Specifically, section ② can be optimized for the width of WG_A, which would include a nonlinear increase as it tapers up to the final width of w_A. Similarly, the lateral separation g between WG_A and WG_B in section ③ can also be optimized, which would result in a faster increasing g as coupling reduces towards the end of the transition. These modified adiabatic couplers can greatly enhance coupling efficiency and extinction ratio while minimizing device length and suppressing fringing in the stop-bands. More generally, improvements may also be possible when using a cross-section similar to the one shown in Fig. 1a, or by using lower index-contrast waveguides, due to reduced interaction of the guided mode with the sidewalls. Waveguide roughness may also contribute to the lower extinction ratio of the short-wavelength filter. Consequently, sidewall passivation, shorter transitions enabled by optimized adiabatic couplers described above, or the use of other waveguiding materials can also enhance the extinction ratio. However, material choices may be limited since phase matching at the cutoff wavelength poses strict restrictions on the material indices."

5. This device is designed with a 100nm minimum waveguide width and spacing. This nano-scale size can be fabricated with E-beam litho process. However, it is usually not allowed for deep-UV litho. Please give a discussion about the feature size. Is it possible to choose larger gap/tip widths for the design, e.g., 150nm or 180nm? How about the performance?

As indicated in the Methods section, the devices were in fact fabricated in a CMOS foundry with 193 nm UV photolithography. On the other hand, as the reviewer suggests, being able to demonstrate similar spectral response with larger gap/tip widths is unquestionably advantageous. In principle, any two coupled waveguides that are phase matched at one wavelength, and phase mismatched at other wavelengths will act as a spectral filter, regardless of the geometry. While it is certainly possible to choose a larger gap g_B, that reduces the effective index of WG_B. For phase matching at the same cutoff, either WG_A would need to be narrower or WG_B would need to have wider individual segments:

- Narrower WG_A: Simulations show that achieving the same cutoff in the C-band with g_B=200 nm requires w_A=285 nm, which is getting quite close to the 220 nm thickness. With such square-like cross-sections, one would expect slightly higher insertion losses at the short wavelength output port due to potential coupling to TM modes.
- Wider individual WG_B segments: It is possible to maintain the same effective index for WG_B by increasing g_B and w_B together. However, while the phase matching condition could still be satisfied, guided modes for both $\lambda < \lambda_C$ and $\lambda > \lambda_C$ would be more strongly confined in WG_B's core segments, farther away from WG_A. Although it may be possible to achieve similar flat-top and low-loss performance, due to the stronger confinement, separating the shorter wavelength mode ($\lambda < \lambda_C$) in section ③ of Fig. 3a would require an exponentially longer transition, resulting in a much larger device footprint. This may be mitigated with the use of optimized adiabatic transitions as we discussed in our response to comment #4 above (also in paragraph 2 of the Discussion).

We added these details in paragraph 2 of the "Adiabatic coupler design" section.

6. It will be helpful for readers if the authors show the simulated light propagation in the designed device.
Upon the reviewer's suggestion, we added a new figure (Figure 4) showing simulated light propagation in the dichroic filter. Due to the large device footprint, we again used the eigenmode expansion method and plotted the electric field magnitude at wavelengths from 1300 nm to 2800 nm. These simulations confirm that the input stays in the fundamental TE mode as it propagates through the designed couplers, and is routed to the corresponding output port throughout more than an octave. From these simulations, we conclude the following:

"Fig. 4a-b show that the extinction ratio at short wavelengths is limited by L_2, as majority of this short wavelength input couples to WG_A in section ②. This is in agreement with the inverse wavelength dependence of κ, confirming the need for a significantly larger L_2 for the same $\int_{L_1}^{L_1+L_2} \kappa\, dz$ at shorter wavelengths. On the other hand, the extinction ratio for wavelengths longer than the cutoff is limited by the final lateral separation of the two output waveguides. Fig. 4g-i show the guided TE mode progressively widening with wavelength and increasing the amount of power coupled to WG_A. With the above choices of lengths, widths, and gaps, the simulation results show the octave-spanning bandwidth of the proposed filter from 1300 nm to over 2800 nm."

The above explanations and interpretations are now added in paragraph 3 of the "Adiabatic coupler design" section. Since this initial demonstration, we also designed similar devices with longer transitions (and a larger resulting footprint) to operate at wavelengths down to 1μm, where the silicon absorption starts becoming significant.

Reviewer #2 (Remarks to the Author):

The paper entitled "Transmissive, Silicon Photonic Dichroic Filters with Spectrally Selective Waveguides," By E. Magden et. al presents the use of waveguides for wavelength selectivity. These serve as single wavelength cutoff filters. The work is interesting and sound and thorough, providing theory, experimental verification. However, the performance metric they use of 2.82dB/nm roll-off is not particularly impressive even within some of the devices cited in Refs. 19-29. Other types of filters which also utilize coupled mode theory in their design and description have been demonstrated rather widely with better roll-off figures. Though not described as dichroic filters, these other filters also serve a similar purpose, which is wavelength selection. Examples of such filters can be broadly classified as mirrors, AWGs, add/drop filters, photonic crystals, wavelength division multiplexers, mode division multiplexers, Bragg gratings (Refs. 19-29 in the manuscript and the following):

Y. Wang et. al, "Ultra-broadband compact and high-reflectivity circular Bragg grating mirror based on 220nm silicon-on-insulator platform," Opt. Express 25(2017). – Few hundred nanometer stopband
J. Foresi et. Al, "Photonic-bandgap microcavities in optical waveguides," Nature 390 (1997) – 300nm stopband

One possible argument in favor of the present device could be that the design presented is a pure cutoff filter which rejects anything outside of the cutoff wavelength, rather than possessing just a limited range of rejected or transmitted wavelengths. However, there are filters with transmission or rejection bandwidths of hundreds of nanometers, which make them functionally similar to the dichroic filters in this work for most applications. Photonic crystals, AWGs, or grating based filters can also be designed to be non-FSR limited with sufficiently large bandwidth to essentially behave as quasi-single wavelength cutoff filters.

The reviewer correctly points out that many filters with sharper roll-offs than demonstrated with this specific device exist in various integrated platforms. Clearly for photonic crystal, ring/disk resonator, AWG, or grating based filters with high quality factors, the filter transmission at the resonances changes dramatically within a fraction of a nanometer. In fact, such devices are commonly used as intra-band add/drop filters in wavelength-division and mode-division applications. Fundamentally however, none of the listed devices can really be classified as what one might call a true "optical low-pass/high-pass filter" that can be used for ultra-broadband optical functionalities. Even the ubiquitous free-space dichroic filters typically have a few hundred nanometer-wide stop or pass-bands. The analysis and performance presented here describes and demonstrates filters whose theoretical bandwidths are only limited by fundamental limits of material absorption. As such, depending on the materials and fabrication processes, and how well the adiabatic couplers are designed, the inherently flat passbands here can be as wide as octaves. This represents the first equivalent of the commonly known RC filter in the field of integrated optics. So far, comparable devices have shown either only a wide bandwidth [48-51, 53, 54], or only a relatively sharp roll-off [52].

We added these explanations in the introduction to better motivate the presented device, and also revised the rest of the introduction according to the reviewer's other suggestions (more details below after each comment).

At the 2100nm wavelength, the transmission bandwidth demonstrated by the authors spans about 200nm. Ref. 2 above is a photonic-bandgap microcavity with a bandwidth of 300nm which is somewhat larger. One suggestion is for the authors to include some simulation results on the potential wavelength span that the long pass and short pass regions can possess, as it is not always straightforward to characterize due to the need for multiple broadband sources.

According to the reviewer's suggestion, we performed eigenmode expansion simulations at wavelengths ranging from 1300 nm to 2800 nm within which the filter shows reliable operation with good performance. Reviewer #1 also had the same suggestion above. As such, we added a new figure (Figure 4) plotting these simulated results showing the octave-spanning simulated operation bandwidth. These results, together with the low-loss flat-top transmissions now clearly illustrate a specific advantage of the on-chip dichroic filter in comparison to previously demonstrated devices. We also made the following observations with this data, and added them in paragraph 3 of the "Adiabatic coupler design" section:

<u>Copied from our response to reviewer #1 above:</u> "Fig. 4a-b show that the extinction ratio at short wavelengths is limited by L_2, as majority of this short wavelength input couples to WG_A in section ②. This is in agreement with the inverse wavelength dependence of κ, confirming the need for a significantly larger L_2 for the same $\int_{L_1}^{L_1+L_2} \kappa \, dz$ at shorter wavelengths. On the other hand, the extinction ratio for wavelengths longer than the cutoff is limited by the final lateral separation of the two output waveguides. Fig. 4g-i show the guided TE mode progressively widening with wavelength and increasing the amount of power coupled to WG_A. With the above choices of lengths, widths, and gaps, the simulation results show the octave-spanning bandwidth of the proposed filter from 1300 nm to over 2800 nm."

Since this initial demonstration, we also designed similar devices with longer transitions (and a larger resulting footprint) to operate at wavelengths as short as 1μm, where the silicon absorption starts becoming significant.

The narrative within the introduction needs improvement. For example:

1) "Many applications in wavelength division multiplexing (WDM) require sharper cutoffs for more efficient utilization of the available spectrum." – WDM in silicon photonics widely demonstrate better cutoffs already, and WDM applications would benefit from actual wavelength selectivity within a narrow range and not a single cutoff.

2) The same can be said of this sentence, "However, narrow pass-bands or limited operation bandwidths present challenges in ultra-wideband photonics as well as WDM applications where

many channels have to be squeezed into a narrow spectrum," where WDM applications typically adhere to the 100GHz or 200GHz standard and would need spectral selectivity over a limited wavelength range rather than a single wavelength cutoff. What is left as a potential application is mentioned as ultra-wideband photonics, but the authors do not give good examples of this. Consequently, the authors could have written a much stronger introduction and found a much better problem statement.

The reviewer has very good points here. As they said, the presented device does not aim to compete with more conventional WDM technologies that would benefit from spectral selectivity over a limited wavelength range. Upon their suggestion, we removed the above referenced statements from the introduction. We revised our problem statement by focusing instead on the applications of such a wideband device in ultra-wideband integrated photonics. We motivated the need for a broadband and efficiently selective filter in regards to recent developments in on-chip supercontinuum generation [14-18] as well as second/high harmonic generation [19-23]. We emphasized these broadband phenomena for the development and scalability of on-chip applications in f-2f [24-26] or f-3f [17] interferometry, spectroscopy [27,28], or wideband WDM [29,30] that require efficient handling of optical signals that are hundreds of nanometers wide. This puts the presented dichroic filter in better perspective for enabling full integration of the above applications, by bringing broadband, efficient wavelength selectivity to integrated systems. These details are now found in the first paragraph of the introduction.

In its present form, I do not recommend publication in Nature Communications as the advancement over the performance of existing devices is not significant, and this has not been well articulated. In particular, the way it is written to emphasize the roll-off value (2.82dB/nm) which is not spectacular in the domain of silicon-based wavelength filters. Having said that, if the authors can demonstrate some other new metrics or functions associated with their devices that are significantly better than existing filters, it could warrant reconsideration for publication Nature Communications.

As indicated with the changes above, after significant revisions to the introduction, we now believe the need for an ultra-wideband, yet efficiently selective filter is stated much better. Simulation of the octave-wide bandwidth in the newly added Figure 4 also emphasizes this unique advantage of the device, and clearly explains how it addresses the ultra-wideband integrated filtering needs. We do not completely de-emphasize the sharp roll-off, as this is important for low-loss filtering operation near the cutoff, and is one of the record metrics of the presented device in the class of adiabatically coupled wavelength filters. However, the revised introduction now focuses on the need for a device combining octave-wide bandwidth, flat-top transmissions, efficient wavelength selectivity, and inherently low-loss operation. With the indicated changes, we now believe the revised motivation, problem statement, and simulation results better convey the need for and the performance of the presented device.

Reviewer #3 (Remarks to the Author):

The paper presents a novel approach to creating integrated photonic spectral filters, with highly desirable high-pass & low-pass cut-off filters, with very sharp roll-offs both in predictions and experimental results. If the problems can be addressed, I expect that it may have a large impact in the field, where many applications of this idea can be applied to.

• Specifically, the major limitation is that the extinction ratio is only 10 dB for the 1550 nm devices, where typically > 30 dB is required for practical applications, including the ones mentioned in the discussion section. At minimum, the manuscript should provide a design (not necessarily fabricated) that solves this issue.

The reviewer is pointing out that the extinction ratio needs to be addressed, at least by providing an improved design. We added a new paragraph about this in the Discussion (paragraph 2), and provided detailed explanations on how to improve the extinction in future devices:

Copied from our response to reviewer #1 above: "In theory, the spectrally-selective waveguide cross-section can provide much larger extinction ratios than measured with the fabricated devices, as predicted in Fig 2c. From a design perspective, the measured extinction is determined by how effectively the input is evolved to the fundamental TE mode at the output. This explains the improved extinction ratio for the 2100 nm filters, due to their longer adiabatic transitions made necessary by their wider waveguides. The extinction ratio can be improved by replacing the linearly tapered adiabatic sections ② and ③ with transitions optimized for a constant adiabaticity measure $\left\langle \psi_A \middle| \frac{\partial}{\partial p} \middle| \psi_B \right\rangle / \delta$ for a given parameter p such as the waveguide widths or gaps [57, 68]. Specifically, section ② can be optimized for the width of WG_A, which would include a nonlinear increase as it tapers up to the final width of w_A. Similarly, the lateral separation g between WG_A and WG_B in section ③ can also be optimized, which would result in a faster increasing g as coupling reduces towards the end of the transition. These modified adiabatic couplers can greatly enhance coupling efficiency and extinction ratio while minimizing device length and suppressing fringing in the stop-bands. More generally, improvements may also be possible when using a cross-section similar to the one shown in Fig. 1a, or by using lower index-contrast waveguides, due to reduced interaction of the guided mode with the sidewalls. Waveguide roughness may also contribute to the lower extinction ratio of the short-wavelength filter. Consequently, sidewall passivation, shorter transitions enabled by optimized adiabatic couplers described above, or the use of other waveguiding materials can also enhance the extinction ratio. However, material choices may be limited since phase matching at the cutoff wavelength poses strict restrictions on the material indices."

• There is some similarity of this work to previous publications, specifically in regards to Fig 1a/b, where WGb is a sub-wavelength waveguide (along the propagation direction, versus in the transverse direction), and where the concept of phase matching between a solid waveguide and a sub-wavelength waveguide is used. The authors should refer their discussion on phase matching

of dissimilar waveguides to previous publications. The following was a directional coupler-based designs, rather than adiabatic, hence is not the same device as this submission: https://www.osapublishing.org/ol/fulltext.cfm?uri=ol-39-24-6931&id=306213. And there are other works on polarization conversion using strip to SWG converters.

The reviewer is correct. The idea of using a high-index material to mimic a lower-index material is similar to a subwavelength grating, and also to some other asymmetric waveguide couplers. As suggested (also by reviewer #1), we included a summary of previous work in various types of asymmetric couplers including those made with sub-wavelength gratings. We added the following summary in the 3rd paragraph of the introduction:

Copied from our response to reviewer #1 above: "Recently, various asymmetric couplers have been used in on-chip polarization or mode-division handling. Coupling between fundamental/higher-order modes [36,37], transverse-electric/transverse-magnetic modes [38-40], or combinations of higher-order and cross-polarization modes [41,42] have been demonstrated. Waveguide asymmetry has also been achieved with the use of sub-wavelength gratings, where the refractive index is periodically modulated in the direction of propagation [43,44] to create fiber-to-chip couplers [45], waveguide crossings [46], and polarization splitters [47] … Similar asymmetric Y-junctions have also been demonstrated in the past for wavelength separation, with the use of different waveguiding materials [50-52] or sub-wavelength gratings [53,54]. …"

In addition to this summary in the introduction, we referred our phase matching discussion in our first Results section to the work we summarized and referenced in the introduction. We identified the key similarities and differences, and added the following explanation in paragraph 2 of page 4:

Copied from our response to reviewer #1 above: "This idea of mimicking a continuous waveguide using segments smaller than the wavelength is similar to a sub-wavelength grating. The difference here is that instead of using the grating duty cycle to create dissimilar waveguides [47,54], we independently engineer the effective and group indices with greater flexibility by controlling the widths and separations of individual segments. Unlike many asymmetric couplers [36-42], the phase matching condition here is satisfied between the fundamental TE modes of the two waveguides, instead of between TE/TM or fundamental/higher-order modes. As such, for the proposed designs, the group index difference is instrumental for realizing phase mismatch at wavelengths outside of the cutoff, and therefore is key for a spectrally selective cross-section."

• The device is highly sensitive to fabrication variations (width, thickness of waveguides). How does it compare to other approaches (AWG, Bragg gratings, contra-directional couplers, etc)?

The reviewer calls attention to the fact that the waveguide effective indices, and hence the phase matching point (the cutoff wavelength), are sensitive to fabrication variations. A similar question was raised by reviewer #1, and we added to following comparison in paragraph 5 of the "Characterization and analysis of filter response" section:

Copied from our response to reviewer #1 above: “For both sets of filters, simulated and measured cutoff wavelengths are listed in Table 1, and show good agreement. The reason behind the slight mismatch is sensitivity of effective indices and the resulting phase-matching point to fabrication variations, similar to many other silicon photonic devices. This is an inherent result of large derivatives of the propagation constants with respect to waveguide dimensions, and is a common occurrence in high index contrast platforms such as Si/SiO_2 or SiN/SiO_2. Here, the difference between the simulated and measured cutoff wavelengths is about 5 nm on average, and can be attributed to the photolithography process-induced changes waveguide geometry. Many other devices that rely on phase-matching also exhibit similar discrepancies as previously reported for Bragg gratings [5,6], AWGs [7,8], and contra-directional couplers [9]. For all these devices, precise spectral alignment is typically achieved with thermal tuning, as detailed in the following section. …”

• On Page 5: “How fast the power in the quasi-even mode evolves from WGA to WGB depends on γ’s rate of change with respect to wavelength.” Does “how fast” refer to the time domain, or equivalently the propagation distance? Or with respect to wavelength? This needs clarification / correction. Similar comment for “The speed at which …” a few paragraph later.

The expressions “how fast” and “speed” here are with respect to the wavelength. They are referring to the sharpness of the spectral transition in wavelength, from one output port to the other. They are not referring to behavior in the time-domain or the propagation distance. We have clarified this by modifying the two sentences and making sure to emphasize with respect to wavelength: “The evolution of power in the quasi-even mode from WG_A to WG_B depends on γ’s rate of change with respect to wavelength” and “The spectral change in $|T_A|$ towards 1 or 0 on either side of the cutoff depends on how fast the magnitude of γ can grow with respect to wavelength.”

• The discrepancy between the simulated versus measured thermo-optic tuning coefficients is almost 20%, which is a much larger error than the cut-off wavelength discrepancy (5 nm at 1550) which is only 0.3%. Yet, the same explanation is given for both discrepancies. Clearly there much be more to the story than just a slight difference in the fabricated thicknesses and widths. More investigation and explanation is required.

The reviewer is asking for a more detailed investigation of the discrepancy between the simulated and experimental values of the thermal tuning efficiency. After careful consideration of the waveguide geometry, we conclude that the simulations can be improved by including both the thermo-optic and thermal expansion effects in the studied waveguides. To this end, we performed full simulations of the effective indices taking into account the thermo-optic coefficients as well as the coefficients of thermal expansion for Si and SiO_2. The results indicate that the previously neglected thermal expansion plays an important role in correctly estimating the filter cutoff wavelength. Specifically, the new simulations with thermal expansion more accurately capture the uneven shifts in the effective indices for WG_A and WG_B, and can more precisely predict their

difference. With increasing temperature, compared to the previous simulation, a slightly larger positive shift in the propagation constant of WG_A (β_A) is estimated. This larger thermally induced shift indicates a larger increase in confinement of ψ_A in the strip waveguide core, compared to the confinement increase of slot mode ψ_B in WG_B's Si segments. By including this thermal dependence of waveguide widths, gaps, and the layer height, we have now shown a simulated cutoff shift of -12.8 GHz/C, which is closer to the that the measured shift of -14.4GHz/C.

The thermo-optic and thermal expansion coefficients used here are $dn_{Si}/dT = 1.86 \times 10^{-4}$ K^{-1} [69], $dn_{SiO2}/dT = 0.95 \times 10^{-5}$ K^{-1} [61], $\alpha_{Si} = 2.61 \times 10^{-5}$ K^{-1} [70], and $\alpha_{SiO2} \approx 5 \times 10^{-7}$ K^{-1} [62]. The simulation result is given in the updated Fig. 6a where we plotted $\delta = (\beta_A - \beta_B)/2$, the mismatch between the propagation constants, as a function of wavelength and temperature. The (wavelength, temperature) pairs at which $\delta = 0$ are traced with the dashed line, showing the thermal dependence of the cutoff with a slope -12.8 GHz/C.

Despite the improved estimation, the simulation and experimental results do not match perfectly. There may be multiple factors contributing to this mismatch: Apart from the fabrication induced changes in the waveguide geometry, the thermal properties (including thermo-optic and thermal expansion coefficients) of the SiO_2 cladding are dependent on the specific conditions of the PECVD process. Any difference from the literature values used here contributes to the mismatch of the thermal tuning efficiency. Additionally, as the thermo-electric cooler controls only the substrate temperature, the thermal impedance of the 2 μm buried oxide may result in the waveguide and substrate temperatures being different. The section titled "Thermal tunability of filter cutoff" has been modified accordingly, and these explanations have been added in paragraph 2.

• The transmission spectra of the measured devices should be overlaid with the simulated devices.

We have plotted the EME simulation results (squares) in the new Fig. 5a and 5b together with the measured transmission spectra (solid lines). We have decided not to plot very similar sets of simulation data 6 more times in Fig. 5d and 5e (3 for each figure), since extinction and roll-off are very similar. The only main difference here is the shift in the cutoff wavelengths, as we have now explained in paragraph 3 of "Characterization and analysis of filter response". We have also summarized these cutoff wavelengths in the new Table 1 for clarity.

• The manuscript is sufficiently detailed for others to be able to reproduce the work.

Thank you for going through the paper in detail, we appreciate your time and efforts.

REVIEWERS' COMMENTS:

Reviewer #1 (Remarks to the Author):

The authors had addressed the reviewer's comments.

Reviewer #2 (Remarks to the Author):

The authors have made significant revisions to the manuscript and strengthened their articulation of the work's novelty. IN particular, the addition of the simulation and a much stronger narrative are good improvements. I recommend publication.

Reviewer #3 (Remarks to the Author):

Thank you for satisfactorily addressing reviewer #2's comments.
